# Subnano Te Cluster in Glass for Efficient Full‐Spectrum Conversion

**DOI:** 10.1002/advs.202303421

**Published:** 2023-10-11

**Authors:** Quan Dong, Ke Zhang, Yupeng Huang, Xu Feng, Tao Yu, Xueliang Li, Jianrong Qiu, Shifeng Zhou

**Affiliations:** ^1^ State Key Laboratory of Luminescent Materials and Devices School of Materials Science and Engineering South China University of Technology Guangdong Provincial Key Laboratory of Fiber Laser Materials and Applied Techniques Guangdong Engineering Technology Research and Development Center of Special Optical Fiber Materials and Devices Guangzhou 510640 China; ^2^ State Key Laboratory of Fluorine and Nitrogen Chemicals Xi'an Modern Chemistry Research Institute Xi'an 710065 China; ^3^ College of Optical Science and Engineering State Key Laboratory of Modern Optical Instrumentation Zhejiang University Hangzhou 310027 China

**Keywords:** cluster engineering, NIR luminescence, optical active materials, Te‐doped

## Abstract

Broadband near‐infrared (NIR) photonic materials have wide applications. Although extensive studies on rare‐earth, transition‐metal, and even semiconductor‐activated materials have enabled the development of a rich NIR material pool, developing broadband and efficient photonic candidates covering the NIR I and II regions from 750 to 1500 nm has been met with limited success. Here, it is reported that a subnano Te cluster with a characteristic configuration different from that of the ion state may fill the aforementioned gap. Further, a strategy is proposed for the in situ generation and stabilization of Te clusters by tuning the cluster evolution in glass. A novel active photonic glass embedded with a Te cluster is fabricated; it exhibits intense and broadband short‐wave NIR luminescence with a central wavelength at 1030 nm and a bandwidth exceeding 330 nm. Interestingly, the glass exhibited a full visible‐spectrum conversion ability from 300 to 800 nm. The application of this unique broadband excitation feature for night vision and tissue penetration is demonstrated using a smartphone as the excitation source. These findings demonstrate a fundamental principle of cluster design in glass for creating new properties and provide a new direction for developing novel cluster‐derived functional composite materials.

## Graphical abstract

The strategy for in situ generation and stabilization of subnano Te cluster in glass is proposed. The photonic glass embedded with Te cluster exhibits unique short‐wave near‐infrared (NIR) luminescence characteristics and full visible‐spectrum conversion ability. The constructed Te cluster‐activated photonic glass is further demonstrated for NIR imaging and broadband optical amplification.

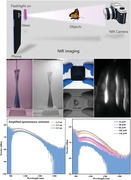

## 1 Introduction

The application of near‐infrared (NIR) luminescent materials has enabled a deeper understanding of natural phenomena, providing new insights in various fields such as optical communication, bioscience, and energy.^[^ ^1^ ^]^ In particular, the short‐wave NIR band, which covers two biological windows (NIR I region: 750–900 nm and NIR II region: 1000–1700 nm) and the entire communication band (1260–1675 nm), has attracted significant attention in recent years.^[^ ^2^ ^]^ On one hand, operating at the short‐wavelength edge of the extended window (≈1200 nm) can provide denser wavelength‐division multiplexing systems that can significantly increase communication capacity.^[^ ^3^ ^]^ On the other hand, higher‐resolution NIR imaging sets more demanding requirements on the coverage range of NIR‐luminescent materials. Furthermore, photonic materials that can effectively transform full visible light into ultrabroadband NIR emissions are an important step forward in advancing the construction of smart optical devices to meet the requirements of energy saving and environmental protection. Driven by these significant applications, substantial efforts have been focused in the past decades in developing novel NIR‐luminescent materials. Star dopants, such as rare‐earth (including Nd^3+^, Yb^3+^, and Er^3+^) and transition‐metal ions (including Cr^3+^, Mn^2+^, and Ni^2+^), have gained considerable attention owing to their abundance of energy‐level configurations.^[^ ^4^ ^]^ However, the spin‐forbidden *f‐f* transition of rare‐earth ions presents virtually no absorption in the visible region and an extremely narrow emission bandwidth (< 70 nm). In addition, the *d‐d* transitions of transition metal ions such as Cr^3+^ and Mn^2+^ are limited by their intrinsic energy levels and are strongly dependent on the crystal field environment, resulting in their main emission peaks and bandwidths being usually less than 850 and 200 nm, respectively.^[^ ^5^ ^]^ Thus, despite considerable efforts, progress in the search for robust NIR luminescent materials with efficient full‐spectrum conversion capabilities is limited by the inherent physical properties of luminescent centers.

**Figure 1:**
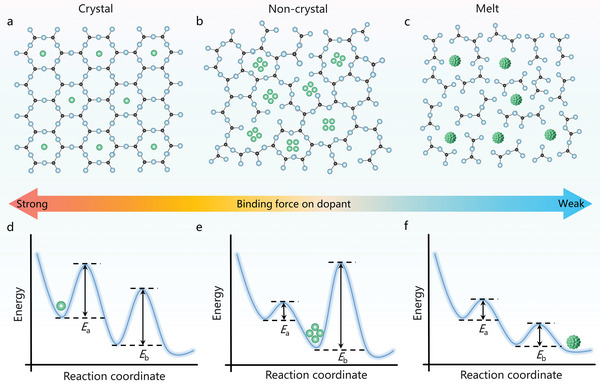
Schematic representation of the structure‐dependent aggregation state evolution. Schematic of the aggregation state of dopants in a) crystal, b) non‐crystal, and c) melt. The middle part illustrates the strength of the binding force on the dopant in three different local structures. d–f) Schematic illustrating the qualitative energetics of the aggregation state evolution of dopants in different structures as well as the resulting products.

A cluster is a stable aggregate state that is distinct from ions,^[^ ^6^ ^]^ and this particular configuration may fill the gap that cannot be reached by traditional rare‐earth and transition metal ions. The physical and chemical properties of clusters vary with the number of atoms they contain and their valence state.^[^ ^7^ ^]^ This leads to abundant energy‐level configurations, which result in unique luminescence potentials. However, clusters, particularly those at the subnano scale, are in an intermediate state between atoms and larger‐sized nanoparticles and are extremely unstable. The stabilization of optically active subnano clusters in condensed matter is a key issue for practical applications in photonics. We attempted to systematically analyze the host‐structure‐dependent dopant state (**Figure** **1**).^[^ ^8^ ^]^ In the general crystal structure, the ions are tightly and orderly arranged according to crystallography theory. Owing to their dense regular structure and strong binding force, external dopants usually replace matrix cations or occupy interstitial sites in the form of ions, rendering the formation of a cluster structure difficult (Figure 1a). For a melt or solution, which is regarded as another type of typical condensed matter, the component structural units are highly discrete. In this framework, the introduced dopant exhibits a strong tendency to aggregate and nucleate. The cluster structures can only exist in a short‐lived transition state and easily form larger nanoparticles (Figure 1c). Based on the aforementioned facts, we expected that the rational control of the structural configuration of the host would help stabilize the intermediate cluster state. To verify this hypothesis, we focused on a glass matrix that belonged to the non‐crystalline state and was characterized by a complex and asymmetric microstructure. This matrix usually contains multimembered rings of various shapes, particularly in multicomponent glasses.^[^ ^9^ ^]^ Figure 1a–c schematically present the structural evolution from crystal–glass–melt, and Figure 1d–f illustrates the difference in the potential barrier that must be overcome for dopant aggregation state transitions in different environments. The typical structural changes during the crystal–glass–melt evolution result in a gradual weakening of the electrostatic binding energy to the aggregate state and strain energy that drives the dopant toward the transition “junction”,^[^ ^10^ ^]^ thereby lowering the potential barrier height. From this perspective, owing to the medium structural binding force, the unique network structure of glass may enable the stabilization of clusters (Figure 1b).

Tellurium (Te) is a chalcogen element that shares chemical properties similar to those of sulfur and selenium. It can form cluster structures consisting of rings, chains, and helices.^[^ ^11^ ^]^ Extensive research has reported the generation of NIR luminescence from Te in glasses.^[^ ^12^ ^]^ However, these materials face two unresolved scientific challenges that significantly hinder their potential for further advancement in photonic applications. One is that the NIR luminescence mechanism of Te remains unclear, and the other is the stabilization of active Te clusters in glasses to achieve highly efficient luminescence.

In this work, we demonstrate the successful construction of novel NIR‐active materials by stabilizing subnano Te clusters in glass. The microstructure‐dependent aggregation state evolution was systematically analyzed, and a unique subnano Te cluster with a characteristic size of 1 nm was achieved. Importantly, the material exhibits an efficient full‐spectrum conversion capability and ultra‐broadband optical response in the short‐wave NIR region, with a peak at 1030 nm in a typical borate glass system. In addition, broadband‐amplified spontaneous emission (ASE) from the fabricated material was achieved. Furthermore, by simply combining a flashlight of a smartphone with the candidate material, a portable NIR light source device was constructed, and its application in night vision and tissue penetration imaging was demonstrated. These results represent significant progress in the development of NIR photonic materials.

## 2 Results and Discussion

### 2.1 In Situ Precipitation of Te Cluster in Glasses

**Figure 2:**
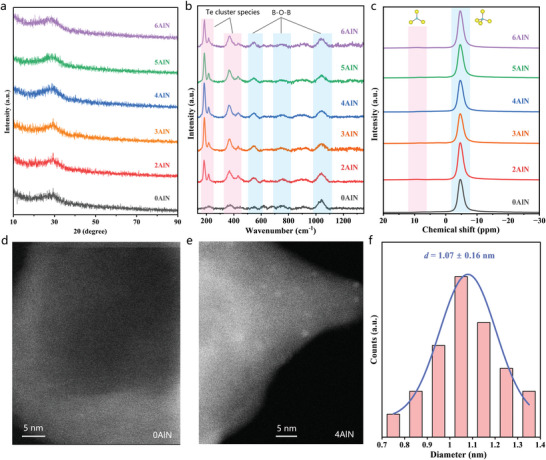
Structural analysis of the Te‐doped borate glasses. a) XRD, b) Raman scattering, and c) ^11^B NMR spectra for samples introduced with different AlN concentrations. d–f) AC‐TEM images of AlN free and 4 mol% AlN added samples.

The microstructures of the (mol%) 40B_2_O_3_‐25K_2_O‐15Cs_2_O‐(20‐*x*/2)Al_2_O_3_‐0.5TeO_2_‐*x*AlN (*x* = 0–6) glass systems were analyzed. Because AlN was used as the precursor, the actual nitrogen contents in glasses with different AlN contents were first measured; the results are shown in Table S1 (Supporting Information). The nitrogen content in the samples retained ≈70 wt.% of the nominal addition after melting. **Figure** **2**a shows the X‐ray diffraction (XRD) patterns of the samples with different AlN contents. All samples were characterized by a diffuse peak at 30°, indicating that the prepared glasses were in an amorphous state. The position and intensity of this diffusion peak do not change with increasing AlN concentration, indicating that the introduction of AlN does not change the crystallinity of the Te‐doped glass. Raman scattering analysis was performed to investigate the bonding features of the glass; the results are shown in Figure 2b. Three distinctive Raman peaks (blue shading) located at ≈550, 750, and 1050 cm^−1^ can be observed, which are attributed to the stretching vibrations of the B─O─B bonds in different structural units.^[^ ^13^ ^]^ Notably, the shape and intensity of these characteristic Raman peaks were highly consistent for all samples (blue shading), indicating that the addition of AlN did not induce obvious changes in the bonding features of the glass. The detailed potential assignments of the Raman peaks are summarized in Table S2 (Supporting Information). One notable phenomenon is the appearance of three extremely strong and sharp Raman peaks at 185, 214, and 370 cm^−1^ (pink shading) in the low wavenumber region once the AlN was introduced. These peaks can be ascribed to the Te‐related centers; the formation of Te nanoparticles can be ruled out because they are generally located in the lower frequency bands of ≈100 cm^−1^.^[^ ^12^, ^14^ ^]^ Particularly, the Raman peak at 214 cm^−1^ originates from the Te_2_ cluster and has been detected in several systems.^[^ ^12^ ^]^ The accurate correlation of the other two Raman peaks (185 and 370 cm^−1^) with the specific Te cluster center remains unclear. The Raman peak at 370 cm^−1^ may be derived from the overtone of the 185 cm^−1^ fundamental frequency. The intensities of these three Te‐related Raman peaks exhibited a strong dependence on the AlN concentration. For example, for the Raman peak at 185 cm^−1^, its intensity first increased and then decreased with increasing AlN content. The strongest Raman scattering occurred at an AlN concentration of 4 mol%, suggesting that excess AlN may induce the formation of other Te cluster species or even nanoparticles. The basic structural units of the host glass were studied using magic‐angle spinning nuclear magnetic resonance (MAS NMR); Figure 2c shows the AlN content‐dependent results. The ^11^B NMR spectra of all glass samples were similar, having a dominant ‐4 ppm spike and a weaker 11 ppm shoulder peak, which can be attributed to the BO_4_ tetrahedron and BO_3_ trihedron, respectively.^[^ ^12^, ^15^ ^]^ The NMR results indicated that the basic structural composition of this glass network was BO_4_ tetrahedra and that the addition of AlN did not significantly change the basic structural units of the glass. These results demonstrate that the introduction of AlN is favorable for the precipitation of high levels of Te‐related clusters directly inside the glass without changing the overall network structure of the glass. These clusters may be able to exhibit extraordinary optical responses.

Spherical‐aberration‐corrected transmission electron microscopy (AC‐TEM) was used to observe the morphology of the Te clusters. Two representative samples, AlN‐free and 4 mol% AlN‐doped, were selected, and their characterization results are presented in Figure 2d–f. No clusters are observed in the AlN‐free sample (Figure 2d). In sharp contrast, bright spots with a regular size of ≈1 nm are clearly observed in the AlN‐containing samples (Figure 2e,f). The observed spots are considered to be Te clusters based on the following facts: First, the formation of Te nanoparticles can be ruled out because they are extremely large in general (approximately tens of nanometers).^[^ ^12^, ^16^ ^]^ Second, the possibility of individual Te atoms or ions can also be dismissed because the size of the atom or ion is very small, usually less than 0.2 nm. Notably, the particles were regularly distributed and relatively uniform in size. This finding implies that they are separate species and that bright spots with larger sizes are likely to be aggregations of these species.

X‐ray photoelectron spectroscopy (XPS) was performed to further understand the changes in the state of Te before and after the introduction of AlN. Two representative samples, AlN‐free and 4 mol% AlN‐doped, were selected, and the results are shown in Figure S1 (Supporting Information). Two prominent peaks are observed in the AlN‐free sample at ≈573 and 583 eV, which are consistent with the peaks in the TeO_2_ crystals and can be attributed to Te 3*d*_5/2_ and Te 3*d*_3/2_, respectively. These results indicate that Te mainly exists in the form of Te^4+^ ions in the AlN‐free sample. However, for the sample with 4 mol% AlN, the two characteristic peaks (573 and 583 eV) were significantly weaker, indicating that the Te^4+^ ions were partially transformed into other species in the glass system. Based on the aforementioned discussion, we can reasonably assume that the introduction of AlN into the glass network significantly enhances the probability of Te transforming from Te^4+^ ions into cluster species.

### 2.2 Theoretical Investigation on the Te Cluster in Glasses

**Figure 3:**
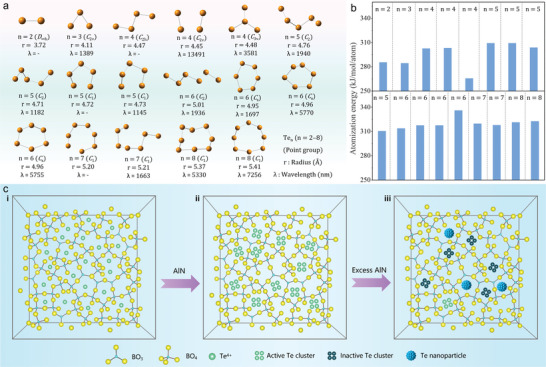
Tracing the origin of Te cluster in glasses. a) A series of thermodynamically stable structures of Te*_n_* (*n* = 2–8) clusters and the corresponding size, point group, and phosphorescence wavelength obtained by theoretical simulations. b) Comparison of the atomization energy of different Te cluster configurations. c) Schematic for the evolution of Te clusters with increasing AlN content.

The physical nature of the Te clusters in the glasses was also studied. Theoretical simulations were employed to identify the thermodynamically stable cluster configurations. Various structural and spectral parameters, including the configuration, physical size, phosphorescence wavelength, and atomization energy, were calculated. As shown in **Figure** **3**a, 17 Te*_n_* (*n* = 2–8) cluster configurations were obtained. When *n* > 3, multiple configurations have the same number of atoms because of the different symmetry elements (point groups). As the number of atoms increases, the radius of the cluster gradually increases. In addition, the results indicate that these cluster species have rich radiative transition potentials and that the corresponding luminescence covers the NIR to mid‐IR regions. The atomization energies of these cluster configurations were compared and are summarized in Figure 3b. With an increase in the number of atoms, the atomization energy of most species increased continuously, except for a few species. Moreover, the atomization energies of clusters with the same atomic number and different configurations were significantly different. Apparently, by integrating the theoretical analysis and AC‐TEM results, the cluster sizes of Te_4_ and Te_8_ can be matched. However, these findings were insufficient to identify the specific cluster configuration.

The physical mechanism of the in‐situ precipitation of Te clusters in glass with the introduction of AlN is summarized and schematically plotted in Figure 3c. The BO_4_ tetrahedral units, which were identified using ^11^B NMR spectroscopy (Figure 2c), were dominant in this borate glass network structure. These BO_4_ units were interconnected to form a complex glass network. In the AlN‐free glass network, the main form of Te was Te^4+^ according to the XPS results (Figure 3c,i). When the highly reducing AlN is introduced into the glass matrix, Te^4+^ is effectively reduced to Te. The accumulation of Te atoms significantly increases their tendency to pack together. In this case, the atomization energy and local structural features of the glass host collaboratively determined the configuration of the precipitated Te cluster (Figure 3c,ii). When the AlN concentration continued to increase, it promoted the formation of larger clusters and nanoparticles (Figure 3c,iii), which was confirmed by AC‐TEM of the sample with 10 mol% AlN (Figure S2, Supporting Information).

### 2.3 Optical Properties of the Te‐Doped Glasses

**Figure 4:**
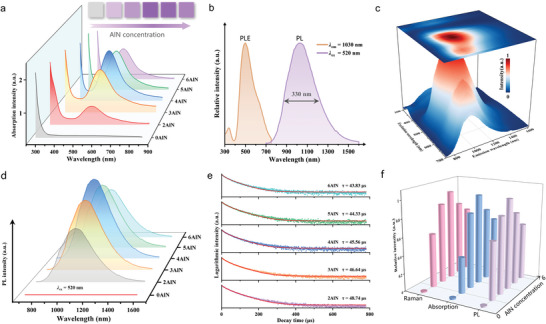
Optical properties of the Te‐doped borate glasses. a) Absorption spectra for samples introduced with different AlN concentrations. The insets show the exterior color of glass samples. b) PLE and PL spectra of the representative sample. c) Dependence of emission spectra on the excitation wavelength. d) PL spectra and e) decay curves of the samples doped with different AlN concentrations. f) Dependence of PL, visible absorption, and Raman intensities of the characteristic Te cluster on AlN concentration.

The optical responses of the Te‐activated series of samples were investigated. **Figure** **4**a shows the absorption spectra of the samples. In the AlN‐free sample, only an interband transition from the host glass occurred, and no characteristic absorption in the visible waveband was observed. With the addition of AlN, all samples exhibited symmetric and broadband absorption covering the entire visible region from 400 to 800 nm, with the central wavelength occurring at 520 nm. The upper inset of Figure 4a shows the appearance of the glass samples; the purple color is consistent with the characteristic absorption. Upon excitation with visible light, the samples with characteristic absorption exhibited intense broadband NIR luminescence, whereas no obvious emission was observed in the AlN‐free sample. Figure 4b shows the representative photoluminescence excitation (PLE) and photoluminescence emission (PL) spectra of Te‐doped samples with 4 mol% AlN. The PLE spectrum exhibited a superbroad excitation band centered at 520 nm, covering the ultraviolet to entire visible region, which matched the absorption spectra well. Under 520 nm excitation, an ultra‐broad symmetric emission band was detected with a maximum at 1030 nm and a full width at half‐maximum (FWHM) of ≈330 nm. Interestingly, this spectral region covers the vast majority of the short‐wave NIR band, far exceeding those of rare‐earth (e.g., Nd^3+^ and Yb^3+^, peak emission ≈1000 nm, FWHM < 70 nm) and transition‐metal ions (e.g., Cr^3+^ and Mn^2+^, peak emission < 900 nm, FWHM < 200 nm). In addition, weak shoulder peaks were observed in both the PLE and PL spectra at 640 and 900 nm, respectively, suggesting that the introduction of AlN induced at least two luminescence centers. To further distinguish between the properties of these two luminescent centers, the dependence of the emission spectra on the excitation wavelength (300–700 nm) was measured (Figure 4c). Under excitation with near‐ultraviolet light, weak emission at 1030 nm was observed. This peak reached a maximum when the excitation wavelength increased to 520 nm. When the excitation wavelength was further increased to 640 nm, a blue shift of the peak emission wavelength and narrowing of the bandwidth were observed, indicating that the luminescence center at 900 nm was dominant. The aforementioned results show that after the introduction of AlN into the Te‐doped system, two types of active luminescent clusters precipitated in situ, corresponding to broadband emission with a peak at 1030 nm and a slightly narrower band at 900 nm. To understand the fluorescence kinetics of this broadband NIR luminescence center, the decay curves of the AlN‐containing samples were recorded under 520 nm excitation, and the results are presented in Figure 4e. All curves were well fitted by a double‐exponential decay function corresponding to the presence of two luminescent centers. With an increase in AlN concentration, the average lifetime decreased monotonically from 48.74 to 43.83 µs. This decrease is mainly associated with the enhanced probability of nonradiative transitions when the Te cluster content is increased.^[^ ^17^ ^]^

To further clarify the origin of the interesting optical response, the relationship between the intensity of the visible characteristic absorption, broadband NIR luminescence, and fingerprint Raman scattering of the Te cluster (185 and 370 cm^−1^) and AlN concentration is summarized in Figure 4f. Interestingly, all of the aforementioned parameters are strongly correlated with each other and show considerable dependence on AlN concentration. They initially increased and then decreased as the AlN content increased, with the optimal AlN concentration being 4 mol%. Further comparison of the experimental phosphorescence wavelengths and theoretical results (Figure 3) revealed that the Te_5_ clusters (*C*_2_ point group, 1182 nm and *C*_1_ point group, 1145 nm) most likely contributed to the observed broadband NIR luminescence. The PL spectra of samples containing different amounts of AlN and TeO_2_ were normalized (Figure S4b,c, Supporting Information). Importantly, AlN did not have a large effect on the spectral shape and peak position, whereas the increase in Te concentration led to a slight red shift of the peak position. This may be due to the entry of the large‐sized Te ions (0.97 Å, CN = 6) to the glass network changing the local environment and affecting the degree of energy level splitting.^[^ ^18^ ^]^ Based on the above results, further optimization of the luminescence properties could be performed. The samples with a Te doping concentration of 0.5 mol% (Figure S4a, Supporting Information) and AlN addition of 4 mol% (Figure 4d) exhibit the best optical performance. The corresponding quantum yields were examined, and the results are shown in Figure S5 (Supporting Information). Under 520 nm excitation, the absorption efficiency reached 68.8%, and the internal quantum efficiency was 15.7%.

### 2.4 Efficient Full Spectrum Conversion Capability of Te‐Doped Glasses

**Figure 5:**
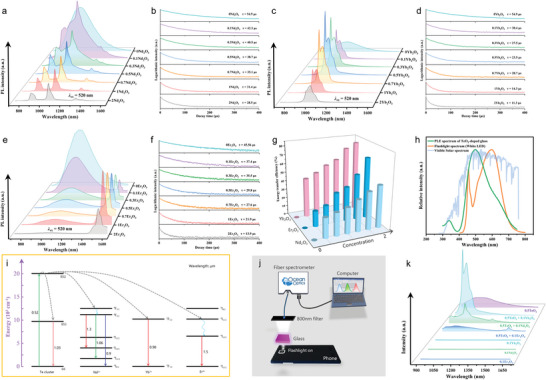
Full‐spectrum conversion capability of Te‐doped and Te‐RE^3+^ co‐doped samples. PL spectra and decay curves of a,b) Te‐Nd^3+^, c,d) Te‐Yb^3+^, and e,f) Te‐Er^3+^ co‐doped samples. g) Comparison of the energy transfer efficiency. h) Comparison of PLE spectra of the Te cluster‐activated glass with the spectra of smartphone flashlight and solar irradiation. i) Schematic diagram of energy transfer for Te‐Nd^3+^, Te‐Yb^3+^, and Te‐Er^3+^. j) Device for investigating the white‐light pumping scheme. k) Detected spectra of various samples pumped by white light.

Trivalent rare‐earth ions (e.g., Er^3+^ and Yb^3+^) have excellent NIR luminescence properties but are often limited by narrow or even no absorption in the visible region. Their strong absorption bands are usually located in the NIR region; the strongest absorption peaks are located near 1000 nm, which prevents them from being efficiently pumped using the most common visible light sources. However, Te‐doped photonic glasses exhibit full visible excitation and ultra‐broadband emission with a peak position at 1030 nm (Figure 3a,b), thus potentially providing new opportunities for the efficient full‐spectrum conversion of rare‐earth ions. To test this, a Te rare‐earth ion sensitization strategy was designed to extend the spectral response of trivalent NIR luminescent rare‐earth ions to the entire visible light spectrum. In general, the construction of sensitization pairs requires the absorption band of the acceptor to overlap with the emission band of the donor.^[^ ^19^ ^]^ Three representative ions, Nd^3+^, Yb^3+^, and Er^3+^, were selected for co‐doping with Te. Three sets of glass samples containing different rare‐earth ion concentrations were prepared, corresponding to Te‐Nd^3+^, Te‐Yb^3+^, and Te‐Er^3+^, where the Te and AlN concentrations were fixed at 0.5 and 4 mol%, respectively. First, the PLE spectra of the Nd^3+^ singly doped and Te‐Nd^3+^ co‐doped samples were compared (Figure S6a, Supporting Information). Nd^3+^ ions have many narrowband excitation peaks, whereas the Te‐Nd^3+^ co‐doped sample exhibits excitation bands for both the Te cluster and Nd^3+^. Subsequently, the PL spectra under 520 nm excitation were compared (Figure S6b, Supporting Information). The Nd^3+^ singly doped sample exhibits three weak characteristic narrowband emission peaks at 900, 1060, and 1320 nm, corresponding to the ^4^ *I*_3/2_–^4^ *I*_9/2_, ^4^ *I*_3/2_–^4^ *I*_11/2_ and ^4^ *I*_3/2_–^4^ *I*_13/2_ transitions of Nd^3+^, respectively. By contrast, the Te‐Nd^3+^ co‐doped sample simultaneously shows the ultra‐broadband emission of the Te cluster and the narrowband emission of Nd^3+^. In addition, the intensity of the Te‐related emission in the co‐doped sample was significantly weaker than that of the Te singly doped sample, and the emission intensity of Nd^3+^ was approximately three times stronger than that of the Nd^3+^ singly doped sample, indicating the existence of a strong energy transfer between the Te active cluster and Nd^3+^. Furthermore, the PL spectra of the samples with different Nd^3+^ concentrations under 520 nm excitation were obtained (**Figure** **5**a). With an increase in the Nd^3+^ doping concentration, the characteristic luminescence of the Te cluster gradually weakened, whereas that of Nd^3+^ first increased and then weakened owing to concentration quenching. To further investigate the energy transfer process, the lifetime of the Te cluster was monitored, and the detected peak position was fixed at 1200 nm to avoid interference from Nd^3+^ luminescence. As shown in Figure 5b, the decay lifetime of the Te luminescent center decreases continuously with increasing Nd^3+^ content, further proving the sensitization of Nd^3+^ by the Te cluster. The energy transfer efficiency (*η*_T_) can be described using the donor decay lifetime and is calculated using Equation 1^[^ ^20^ ^]^:

(1) $$\eta_{T}=1-\frac{\tau_{s}}{\tau_{so}}$$

where *τ_S0_* and *τ_S_* are the decay lifetimes of the donor absence and presence of the acceptor Nd^3+^, respectively. The maximum energy transfer efficiency was estimated to be 48%.

In addition, the sensitization effect of Te clusters on Yb^3+^ was studied. The PLE spectra of the Yb^3+^ singly doped and Te‐Yb^3+^ co‐doped samples were compared (Figure S7a, Supporting Information). When monitored at 1030 nm, the Yb^3+^ singly doped sample exhibits a sharp excitation band at 900 nm. Importantly, the Te‐Yb^3+^ co‐doped sample is characterized by broadband excitation, which covers the entire visible region, indicating energy transfer between the Te active cluster and Yb^3+^. The emission spectra of Yb^3+^ singly doped and co‐doped samples were recorded under 520 nm excitation, and the results are shown in Figure S7b (Supporting Information). The Yb^3+^ singly doped sample did not show any optical response under 520 nm excitation. For the Te‐Yb^3+^ co‐doped sample, an intense Yb^3+^ characteristic emission at 1000 nm (^2^ *F*_5/2_–^2^ *F*_7/2_ transition of Yb^3+^) appeared, and the intensity exceeded that of the Te singly doped sample, demonstrating an efficient energy transfer process from Te active clusters to Yb^3+^. A series of co‐doped samples with different Yb^3+^ concentrations were prepared, and their spectral behavior upon excitation at 520 nm was characterized. As shown in Figure 5c, with increasing Yb^3+^ content, the emission peak of Te sharply decreased, and the characteristic emission of Yb^3+^ was significantly enhanced. In addition to the efficient full‐spectrum conversion capability, another advantage of the Te‐Yb^3+^ sensitization strategy is that it can prevent the unexpected energy loss caused by conventional Yb^3+^ pumping (980 nm) because of the large overlap between the absorption and emission bands of Yb^3+^. To further investigate the energy transfer efficiency between the Te cluster and Yb^3+^, the decay process of the Te cluster in a series of samples was recorded at 1200 nm to eliminate the interference of Yb^3+^ luminescence. The introduction of Yb^3+^ leads to a considerable decrease in the lifetime of the Te cluster from 54.9 to 11.3 µs, far exceeding that in the Te‐Nd^3+^ co‐doped samples (Figure 5d). The energy transfer efficiency of Te‐Yb^3+^ can reach 80%, as calculated using Equation 1, further illustrating the efficient sensitization process between the Te cluster and Yb^3+^.

Furthermore, the sensitization process between Te clusters and Er^3+^ was studied. The PLE spectra of the Er^3+^ singly doped and Te‐Er^3+^ co‐doped samples monitored at 1550 nm are shown in Figure S8a (Supporting Information). Er^3+^ exhibited narrow excitation bands in several regions, including 520 nm. By contrast, the Te‐Er^3+^ co‐doped samples had broad excitation bands in the visible region. The PL spectra of Er^3+^ singly doped and co‐doped samples were recorded under 520 nm excitation (Figure S8b, Supporting Information). A narrow emission band at 1550 nm was detected for Er^3+^ alone, which can be attributed to the ^4^ *I*_13/2_–^4^ *I*_15/2_ transition of Er^3+^. When Te‐Er^3+^ coexisted, in addition to the characteristic emission of Er^3+^, an ultra‐broadband emission of the Te cluster at 1030 nm was observed. Notably, this emission band is significantly suppressed compared to that of the Te singly doped samples, indicating that the co‐doped Er^3+^ consumes most of the excited state energy of the Te cluster. To further demonstrate the existence of energy transfer between the Te cluster and Er^3+^, we moved the excitation wavelength to a longer position to excite only the Te cluster and not Er^3+^ (Figure S8c, Supporting Information). Even when the excitation wavelength was switched to 600 nm, the characteristic emission of Er^3+^ could still be detected, further proving that Er^3+^ can absorb energy from the Te cluster and convert it into a 1550 nm emission. The PL spectra under 520 nm excitation for serial co‐doped samples with different Er^3+^ concentrations were characterized, and the results are shown in Figure 5e. With increasing Er^3+^ content, the PL intensity of the Te cluster decreases significantly, whereas the characteristic emission of Er^3+^ at 1550 nm first increases and then decreases. Figure 5f shows the Er^3+^ doping concentration‐dependent decay lifetime of the Te cluster emission at 1030 nm. Similar to the cases of the Te‐Nd^3+^ and Te‐Yb^3+^ co‐doped samples, the increase in Er^3+^ content led to a sharp decrease in the decay lifetime of the Te cluster, further demonstrating the efficient energy transfer process between the Te cluster and Er^3+^. The maximum energy transfer efficiency was estimated to be 70%.

Figure 5g summarizes a comparison of the energy transfer efficiencies of the Te‐Nd^3+^, Te‐Yb^3+^, and Te‐Er^3+^ co‐doped samples. The results are different and can be explained from the viewpoint of the energy transfer theory.^[^ ^21^ ^]^ The probability of energy transfer between the donor (D) and acceptor (A) can be described using the following formulas:

(2) $$P_{DA}=\frac{3{\bar{h}}^{4}c^{4}}{4\pi n^{4}R_{DA}^{6}}\frac{Q_{A}}{\tau_{D}}\int \frac{\lambda_{D}\left(E\right)\lambda_{A}\left(E\right)}{E^{4}}dE$$

(3) $$\bar{h}=\frac{h}{2\pi }$$

where *P*_DA_ is the probability of energy transfer, *h* is Planck's constant, *c* is the speed of light, *n* is the refractive index of the medium, *R*_DA_ is the distance between the donor and the acceptor in host, and *Q*_A_ is the effective absorption cross section of the acceptor transition, which is associated with the absorption spectrum, *τ*_D_ is the excited state lifetime when the donor exists independently, and $\int \frac{\lambda_{D}\left(E\right)\lambda_{A}\left(E\right)}{E^{4}}dE$ is the integral of the overlapping area between the donor emission and acceptor absorption bands. In this system, the donor is unified as the Te cluster and τ_D_ is fixed. A schematic for *R*_DA_ comparison of the different Te‐RE^3+^ co‐doped systems is shown in Figure S9 (Supporting Information). The Te content was fixed at 0.5 mol% and the Te active clusters were evenly distributed throughout the glass. When the rare‐earth ions were introduced, they were evenly distributed. The *R*_DA_ comparison was based on the same rare‐earth ions content; therefore, the average distance (*R*_DA_) between the Te cluster and the three different types of rare‐earth ions can be considered to be approximately equal. That is, *R*_DA_ (Te‐Er^3+^) ≈ *R*_DA_ (Te‐Nd^3+^) ≈ *R*_DA_ (Te‐Yb^3+^). Therefore, *P*_DA_ is directly proportional to the absorption cross sections and spectral overlapping areas of the three different rare‐earth ions. In other words, the absorption capacity of the acceptor near the emission peak of the donor and the energy transfer efficiency are positively correlated. Comparing the absorption bands of Er^3+^, Yb^3+^, and Nd^3+^ (Figure S10, Supporting Information), Er^3+^ and Yb^3+^, particularly Yb^3+^, have extremely strong absorption near the central emission region of the Te cluster (≈1000 nm), whereas Nd^3+^ only has a small absorption in the sideband region. The significant differences in the energy transfer efficiencies of the samples can be directly attributed to the aforementioned facts. The detailed energy‐transfer processes from the Te cluster to Nd^3+^, Yb^3+^, and Er^3+^ are illustrated in Figure 5i. The electrons in the ground state of the Te cluster can be pumped to the high‐energy excited state (ES2) by visible light and then relaxed to the low‐energy excited state (ES1) through nonradiative transition. Subsequently, the electrons undergo a radiative transition back to the ground state and transfer energy to the rare earth ions. This enables the simultaneous generation of the characteristic emission of the Te cluster and rare‐earth ions.

The as‐prepared Te‐doped photonic glass exhibited full‐spectrum conversion capability from visible light to a broadband NIR waveband. Notably, the excitation spectrum of the material has an extremely large overlap region with the flashlight and solar spectra (Figure 5h). This finding raised a new question: can this photonic glass be pumped by white light or sunlight? A white‐light‐pumping device was built to test this hypothesis. A smartphone flashlight was used as the white‐light source (Figure 5j). The photonic glass samples were placed above the flashlight and covered with an 800 nm ​​long‐pass filter. The optical signal was collected using an NIR fiber spectrometer (900–1700 nm). Figure 5k shows the recorded spectra of various samples. Interestingly, the intense broadband NIR luminescence of the Te cluster was successfully detected under white‐light pumping (purple line). Furthermore, the Te‐Nd^3+^, Te‐Yb^3+^, and Te‐Er^3+^ co‐doped samples can also be effectively pumped by white light, and various characteristic emissions can be observed. In contrast, the samples doped with rare‐earth ions showed no obvious optical response under white‐light pumping. This result is highly interesting because it demonstrates that Te‐doped photonic glass has a robust full‐spectrum conversion capability. Finally, a comparison of the NIR luminescence properties of the Te‐doped glass developed here with those of conventional rare‐earth and transition‐metal ion‐doped systems is summarized in Table S3 (Supporting Information). The Te cluster‐activated photonic glass not only exhibits a bandwidth (≈330 nm) far exceeding that of other types of centers and full visible‐light pumping characteristics but also fills the gap of the lack of ideal activators near 1000 nm.

### 2.5 Photonic Applications of Te Cluster‐Activated Glass

**Figure 6:**
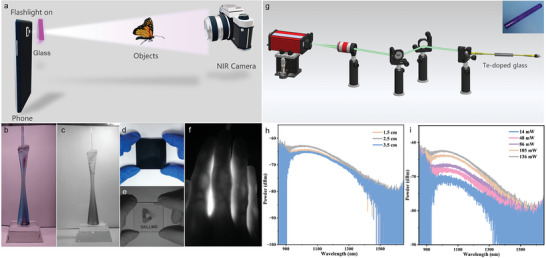
Photonic applications of Te cluster‐activated glass. a) Schematic diagram of NIR imaging. Visible and NIR imaging for b,c) Canton Tower model, d,e) pattern under 800 nm filter, and f) finger. g) Schematic diagram of the optical system for collecting the ASE signals. The inset is an actual photograph of the glass rod. ASE spectra as a function of glass rod length h) and pump power i).

The attractive optical properties of the Te cluster‐activated glass prompted us to explore its photonic applications. The constructed simple NIR light source device can be used for NIR imaging (**Figure** **6**a). Only the Te singly doped sample was used as the light conversion medium. A block glass was attached to the flashlight of the smartphone to generate NIR light, which was then used to illuminate objects. The reflected photons were captured and imaged using a NIR camera.

A model of the Canton Tower was used for this demonstration. Figure 6b shows an image of the model under white fluorescent light, not LED white light, from a smartphone, representing a normal color image. Figure 6c shows the black and white images obtained using the constructed NIR light‐source device. Figure 6b was captured using a normal visible camera, whereas Figure 6c was captured using an NIR camera with the fluorescent light turned off. Even with the fluorescent light off, a full view of the object could still be observed except for the color. To further highlight the difference, an 800 nm long‐pass filter was used to cover the pattern, and a photograph under fluorescent light is shown in Figure 6d. Evidently, the “SKLLMD” character and image were covered by the 800 nm long‐pass filter and completely invisible. However, when the constructed NIR light source device was turned on and the image photographed with an NIR camera, the pattern under the filter was clearly observed; the filter behaved as a transparent glass (Figure 6e). A prominent advantage of the constructed NIR light source device is its excellent tissue‐penetrating imaging capability. To demonstrate this, a finger was placed over the device, and an NIR camera was used to capture the image. As illustrated in Figure 6f, the veins inside the finger can be clearly observed. Figure S11 (Supporting Information) shows the results of using the constructed NIR light source to penetrate chicken breast slices of different thicknesses. The collected NIR photons gradually weaken as the thickness of the chicken breast increases because O─H and C─H in meat tissue have strong absorption in the 960, 1200, and 1400 nm regions.^[^ ^22^ ^]^ The detailed experimental process and results are presented in the Supporting Information. These results not only show that the simple NIR light source devices constructed using the fabricated Te cluster‐activated photonic material facilitates imaging in night vision and shallow tissues but also provides a new opportunity for the development of a new generation of white light pumpable NIR light sources.

Second, the wide coverage range in the NIR region of the Te cluster‐activated glass indicates its potential for broadband optical amplification in telecommunication systems. Glass is malleable and can take various shapes. As shown in Figure 6g, thin glass rods with diameters of 2 mm were fabricated, and their amplified spontaneous emission (ASE) properties were characterized using a self‐built spatial optical system. The light emitted from the 532 nm laser device was collimated by an objective lens and then reflected by two mirrors into a fiber collimator to couple the light into a multimode fiber patch cable. The glass rod was then clamped between the two fiber patch cables using a holder, and the rear end was connected to an optical spectrum analyzer for signal acquisition. In addition, an aluminum film was wrapped around the exterior of the glass rod to keep as little light as possible from leaking out from the sides. Figure 6h shows the ASE spectra recorded for glass rods of different lengths. The ASE spectra covered a wide range of O, E, and S bands in the telecommunications window, indicating its potential for broadband optical amplification. The ASE was strongest through a glass rod length of 2.5 cm; further lengthening may lead to a decrease in the ASE intensity owing to optical losses. Subsequently, the ASE spectra of the 2.5 cm glass rod were further investigated as a function of the pumping power, and the results are shown in Figure 6i. The ASE intensity increased monotonically with increasing pump power, and no obvious saturation was observed, thus demonstrating the potential value of the Te cluster‐activated photonic glass in high‐power device packaging.

## 3 Conclusions

In conclusion, we proposed that subnano Te clusters may present a unique optical response that cannot be achieved by conventional active ions. We demonstrated the successful generation and stabilization of Te clusters by tuning the cluster evolution in glass. The fabricated photonic glass embedded with a Te cluster exhibited intense and broadband short‐wave NIR luminescence with a central wavelength of 1030 nm and bandwidth of more than 330 nm. It also exhibited a full visible‐spectrum conversion ability from 300 to 800 nm. In addition, we demonstrated its application in night vision and tissue penetration by employing a smartphone as the excitation source. Furthermore, we confirmed that the glass can be used to generate intense ASE signals in a broad telecommunication waveband. The new discoveries regarding subnano Te cluster engineering are expected to not only reveal new basic principles of cluster design in amorphous solids but also provide important clues for the development of novel cluster‐derived functional composites for various photonic applications.

## 4 Experimental Section

### Glass Melting

A series of borate glasses with nominal compositions of (mol%) 40B_2_O_3_‐25K_2_O‐15Cs_2_O‐(20‐*x*/2)Al_2_O_3_‐0.5TeO_2_‐*x*AlN (*x* = 0–6) were synthesized via a traditional melt‐quenching route in air. In addition, glasses with different TeO_2_ contents were synthesized to optimize the luminescence properties with TeO_2_ concentrations ranging from 0 to 1 (mol%). A detailed rationale for the choice of this glass component is provided in the Supporting Information (Figure S3, Supporting Information). To understand the chemical state of Te upon addition of excess AlN, a glass sample containing 10 mol% AlN was prepared under the same conditions. To investigate the synergistic luminescence properties of Te and rare‐earth ions, a series of Te‐RE^3+^ co‐doped glasses with compositions of (mol%) 40B_2_O_3_‐25K_2_O‐15Cs_2_O‐18Al_2_O_3_‐0.5TeO_2_‐4AlN‐*y*RE_2_O_3_ (RE = Nd, Yb, and Er, *y* = 0–2) were prepared using the same method. High‐purity reagents were selected as the raw materials. In a typical run, 20 g batches were accurately weighed according to the given stoichiometric ratio, mixed homogenously, placed in alumina crucibles, and finally melted at 1300 °C in a box furnace for 25 min. The melt was poured onto a preheated stainless‐steel plate and pressed using another plate to form solid glasses. Subsequently, the glass samples were annealed at 400 °C to relieve internal stress. Finally, all samples were finely cut and polished to a size of 10 mm × 10 mm × 2 mm for further characterization. In addition, thin glass rods of diameter 2 mm and lengths 1.5–3.5 cm used for ASE characterization were obtained by cutting and polishing the larger bulk glass.

### Material Characterization

The phase compositions of the samples were analyzed using an X’ Pert Pro X‐ray diffractometer (XRD, Holland PANalytical) with Cu–Kα1 radiation (*λ* = 1.5405 Å; cathode voltage, 40 kV; current, 40 mA) in the region–5°–90°. The actual nitrogen content in the glasses was measured using an oxygen nitrogen hydrogen analyzer (American LECO Company, ONH836). The microstructures were studied at the nanometer scale using an FEI Spectra 300 spherical aberration‐corrected transmission electron microscope (Thermo Fisher). Raman spectra were measured using a Raman spectrometer (Renishaw in Via) with a 25 mW Nd: YAG laser at 532 nm. NMR spectra of ^11^B were obtained using a Bruker AVANCE III HD 500 instrument at a frequency of 160.5 MHz. XPS was performed using a photoelectron spectrometer (Kratos, Axis Supra +). The absorption spectra were measured using a UV–visible–NIR spectrophotometer (PerkinElmer, Lambda 900) covering the spectral range of 300–1500 nm. PLE, PL, and luminescence decay spectra were collected on an Edinburgh FLS 920 fluorescence spectrofluorometer (Edinburgh Instruments) equipped with a 450 W Xenon lamp, a liquid‐nitrogen‐cooled photomultiplier (Hamamatsu R550972), and a microsecond flashlamp (*µ*F900). Quantum efficiency was measured using an Edinburgh FLS 1000 fluorescence spectrophotometer with visible and NIR photomultiplier tube detectors. The NIR PL spectra under 808 and 980 nm laser diode excitation were measured using a spectrometer (Zolix, Omni λ3007) equipped with an InGaAs photodetector, an SR830 Stanford lock‐in amplifier, and an SR540 chopper. The emission spectra of the portable devices were collected using an NIR fiber spectrometer (NIRQUEST 512, Ocean Optics, NIR, 900–1700 nm). The optical components used to obtain the ASE spectra, including the objective lens, reflector, and fiber collimator, were purchased from THORLABS. In addition, ASE spectra were measured using an optical spectrum analyzer (OSA, Yokogawa, AQ6370C). All measurements were performed at room temperature. Visible images were captured using a digital camera (Canon 700D). NIR imaging was performed using a modified infrared camera (Canon EOS 80D), the lens of which was covered with an 800 nm filter to block any visible light. The exposure time, aperture size, and focal length were 1/6 s, f/5.6, and 18 mm, respectively. A glass Canton Tower model was used for NIR imaging. The “SKLLMD” character and image pattern was printed on white paper by a printer using black ink. The flashlight spectrum of the smartphone was identified as a white LED (blue chip + YAG:Ce) with a power of less than 1 W. The smartphone model used was HONOR V20, produced by the HONOR Company (HONOR China).

### Theoretical Simulations

The initial structures were built according to a global optimum search using ABCluster2.0^[^ ^23^ ^]^ with the GFN2‐xTB method.^[^ ^24^ ^]^ The geometries of the ground states were optimized at the PBE0/def2‐TZVP level. PBE0^[^ ^25^ ^]^ contained 25% exact exchange. The basis set def2‐TZVP^[^ ^26^ ^]^ considered the effective core potential of Te atoms. The geometries of the clusters are consistent with those shown in the references.^[^ ^11^ ^]^ The radii of the clusters were derived from the volumes enclosed by the 0.001 electrons bohr^−3^ isodensity surface of the electrostatic potentials. To calculate the phosphorescent wavelengths, the geometries of the excited states were optimized at the PBE0/def2‐TZVP level, and the single‐point energies of the ground states were based on the optimized geometries at the same level. The wavelengths were determined from the energy gaps between the excited and ground states. None of the optimized geometries had imaginary frequencies. Computational electronic structures were realized using Gausssian16.^[^ ^27^ ^]^

## Conflict of Interest

The authors declare no conflict of interest.

## Supporting information

Supporting Information

## Data Availability

The data that support the findings of this study are available from the corresponding author upon reasonable request.
