# Supplementary material for: Subnano Te Cluster in Glass for Efficient Full‐Spectrum Conversion
Source: Adv Sci (Weinh). 2023 Oct 11;10(33):2303421. doi: 10.1002/advs.202303421 (PMC10667836; doi:10.1002/advs.202303421)
Supplement: Supplementary file 1 — Supporting Information [file ADVS-10-2303421-s001.pdf]

ADVANCED SCIENCE
Open Access

Supporting Information

for *Adv. Sci.*, DOI 10.1002/advs.202303421

Subnano Te Cluster in Glass for Efficient Full-Spectrum Conversion

*Quan Dong, Ke Zhang, Yupeng Huang, Xu Feng, Tao Yu, Xueliang Li, Jianrong Qiu and Shifeng Zhou**

Supporting Information

Subnano Te cluster in glass for efficient full-spectrum conversion

Quan Dong, Ke Zhang, Yupeng Huang, Xu Feng, Tao Yu, Xueliang Li, Jianrong Qiu, Shifeng Zhou *

Q. Dong, K. Zhang, Y. P. Huang, X. Feng, X. L. Li, S. F. Zhou

State Key Laboratory of Luminescent Materials and Devices, School of Materials Science and Engineering, South China University of Technology. Guangdong Provincial Key Laboratory of Fiber Laser Materials and Applied Techniques, Guangdong Engineering Technology Research and Development Center of Special Optical Fiber Materials and Devices, Guangzhou 510640, China.

E-mail: zhoushifeng@scut.edu.cn

T. Yu

State Key Laboratory of Fluorine and Nitrogen Chemicals, Xi'an Modern Chemistry Research Institute, Xi'an, 710065, China.

J. R. Qiu

College of Optical Science and Engineering, State Key Laboratory of Modern Optical Instrumentation, Zhejiang University, Hangzhou 310027, China.

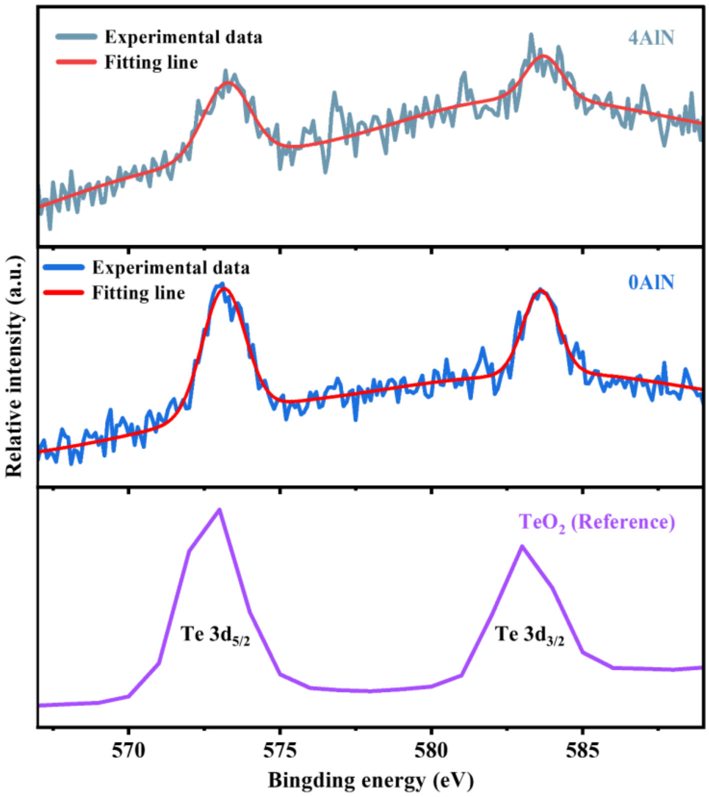

Figure S1. High-resolution Te 3d XPS spectra of AlN free and 4 mol% AlN added samples. The spectrum of TeO_2 was also shown as reference.

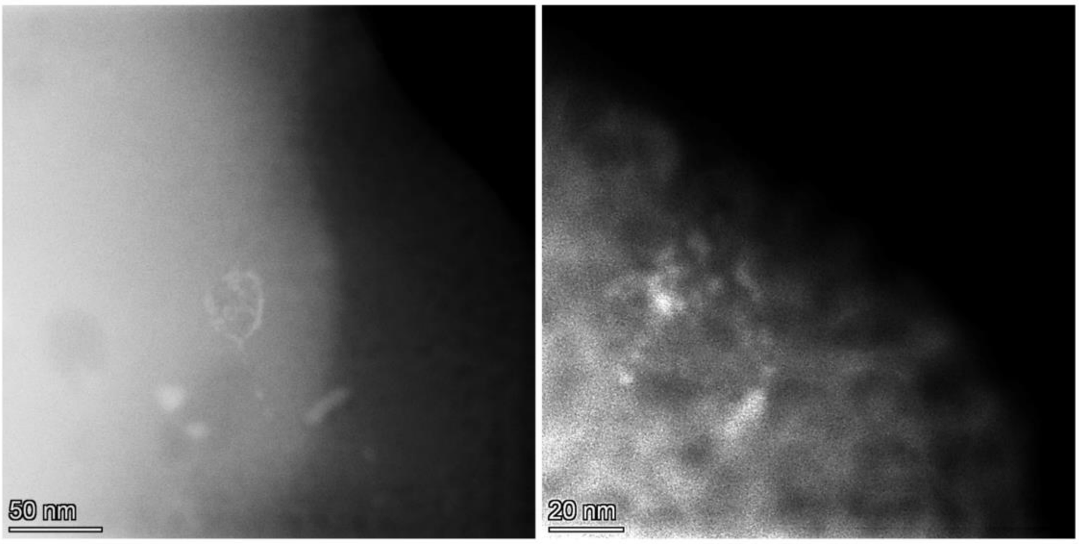

Figure S2. AC-TEM images of the sample with the addition of 10 mol% AlN.

When the concentration of AlN is 10 mol%, the particles size of Te related center is about 3-15 nm. This may be due to the severe aggregation of local Te atoms to form nanoparticles.

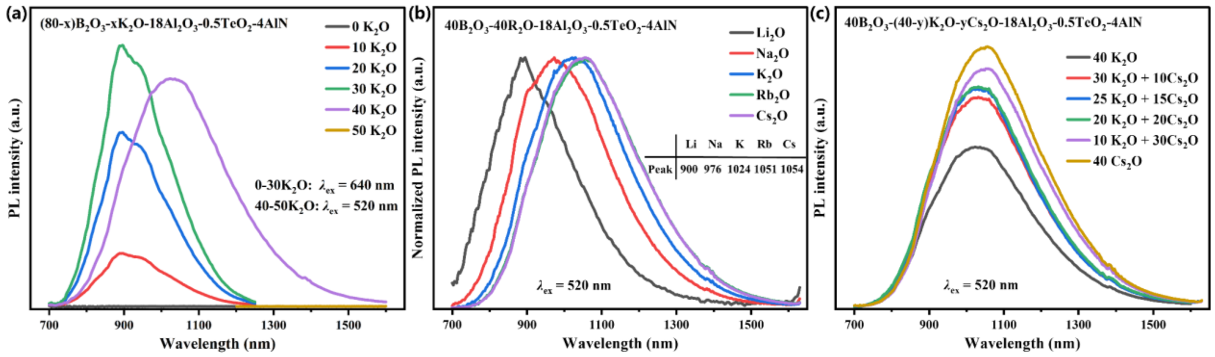

Figure S3. Components dependent luminescence properties. (a) K_2O concentration dependent PL spectra. (b) Alkali metals dependent PL spectra. (c) K_2O/Cs_2O ratio dependent PL spectra.

The changes of K_2O contents and types of alkali metals in the glass lead to significant changes in luminescence performances, suggesting that the glass structure might be changed to promote the precipitation of active Te clusters. A unique ultra-broadband luminescence located at near 1030 nm can be observed in glass samples containing K_2O, Rb_2O and Cs_2O. Unfortunately, these samples exhibit poor stability. In order to improve the glass stability, a mixed-alkali method was employed. It can be found that the $25K_2O + 15Cs_2O$ sample show the best glass stability.

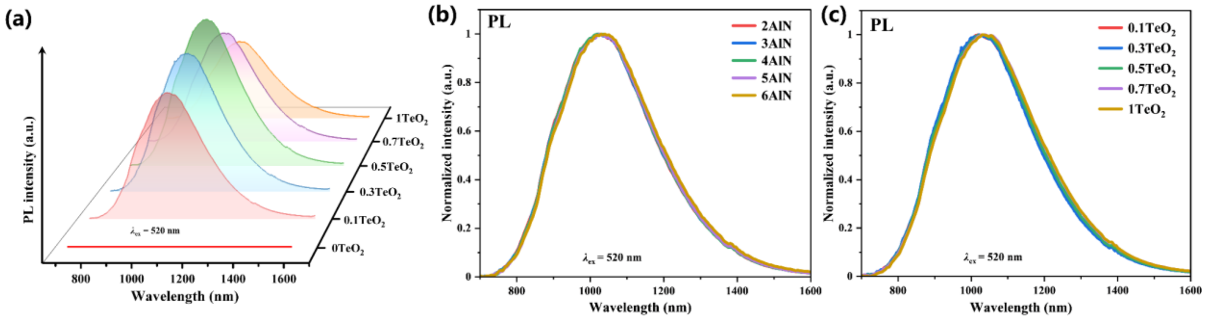

Figure S4. (a) Dependence of PL spectra on TeO_2 concentration. (b, c) Normalized PL spectra of samples doped with different amounts of AlN (b) and TeO_2 (c).

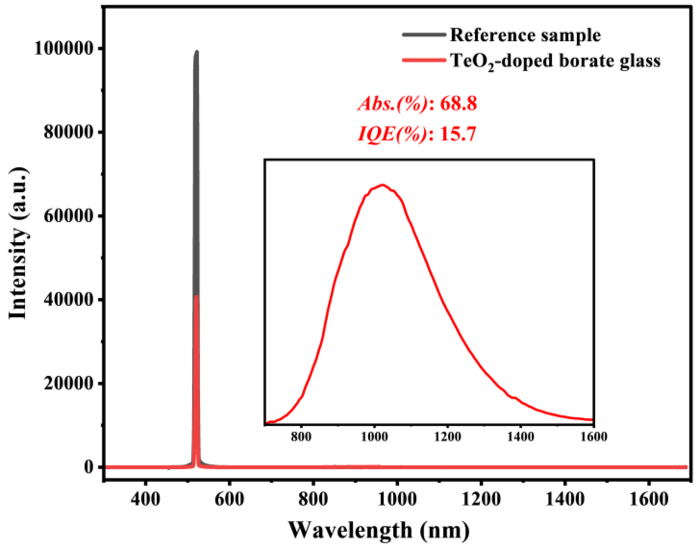

Figure S5. Quantum yields of the 4 mol% AlN added sample under 520 nm excitation.

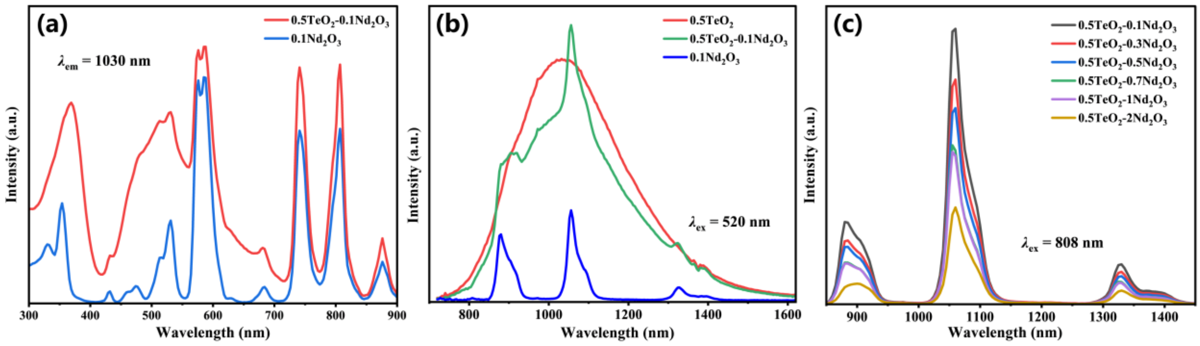

Figure S6. (a) PLE spectra of Nd^{3+} singly doped and Te-Nd^{3+} co-doped samples monitored at 1030 nm. (b) PL spectra of Te, Nd^{3+} singly doped and Te-Nd^{3+} co-doped samples under 520 nm excitation. (c) PL spectra of Te-Nd^{3+} co-doped samples with Nd^{3+} concentration under 808 nm LD excitation.

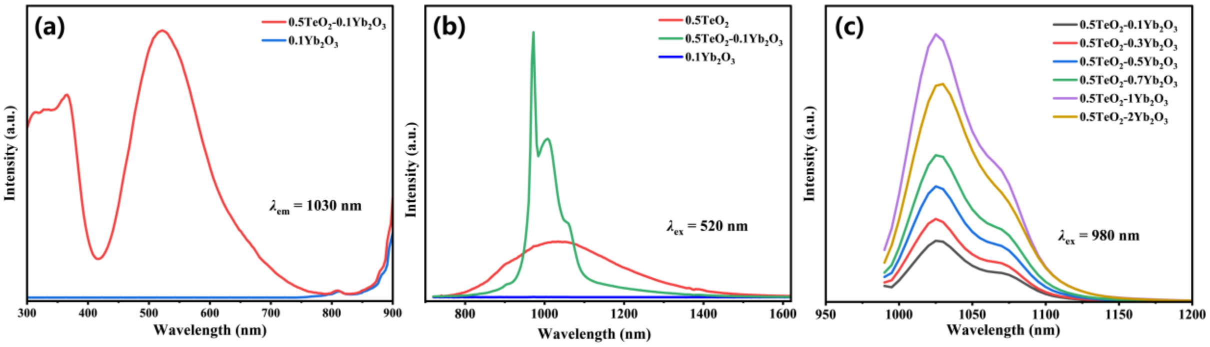

Figure S7. (a) PLE spectra of Yb^{3+} singly doped and Te-Yb^{3+} co-doped samples monitored at 1030 nm. (b) PL spectra of Te, Yb^{3+} singly doped and Te-Yb^{3+} co-doped samples under 520 nm excitation. (c) PL spectra of Te-Yb^{3+} co-doped samples with Yb^{3+} concentration under 980 nm LD excitation.

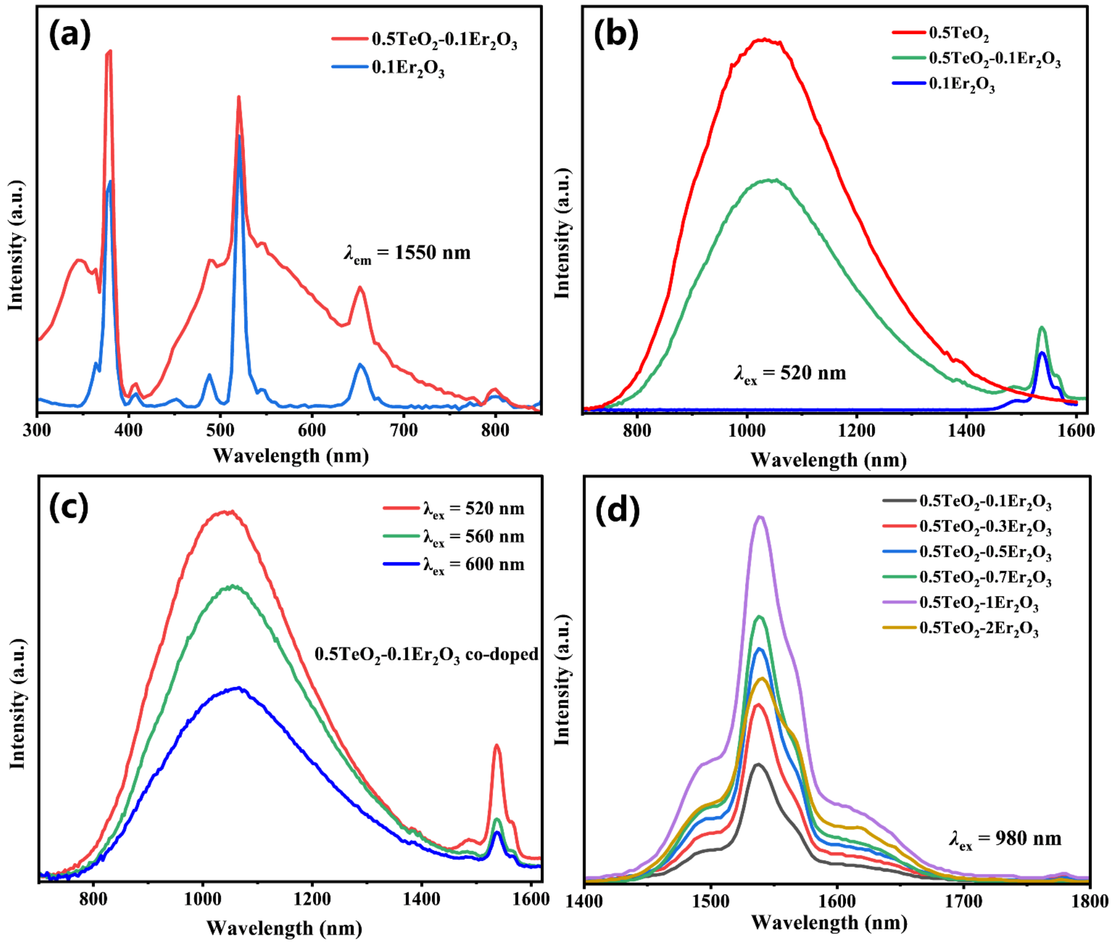

Figure S8. (a) PLE spectra of Er^{3+} singly doped and Te-Er^{3+} co-doped samples monitored at 1550 nm. (b) PL spectra of Te, Er^{3+} singly doped and Te-Er^{3+} co-doped samples under 520 nm excitation. (c) PL of Te-Er^{3+} co-doped representative samples under different excitation wavelengths. (d) PL spectra of Te-Er^{3+} co-doped samples with Er^{3+} concentration under 980 nm LD excitation.

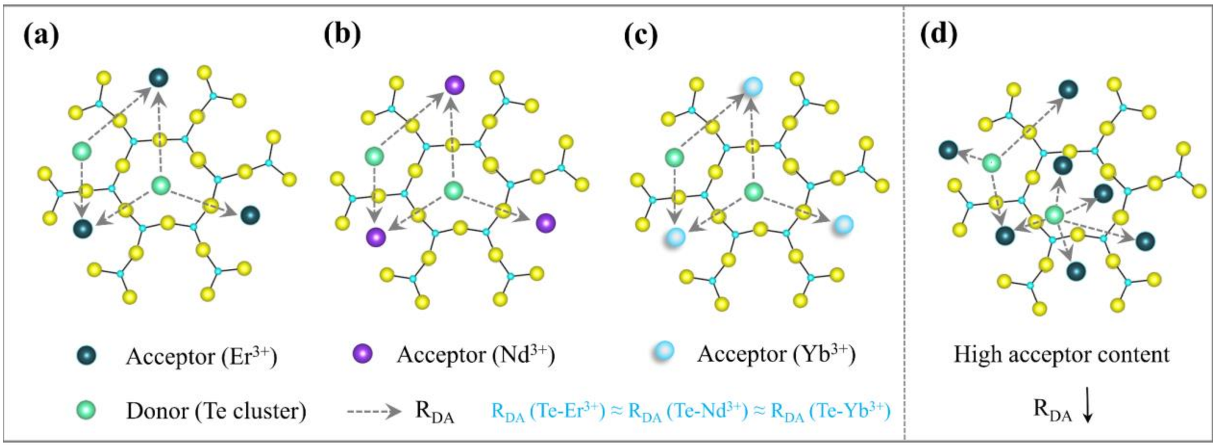

Figure S9. (a-c) Schematic drawing for illustration of R_{DA} in different Te-RE^{3+} co-doped systems. (d) Changes of R_{DA} with the increased RE^{3+} content.

The R_{DA} will gradually decrease when the content of rare earth ions (acceptors) gradually increases (Figure S9d). According to formula (2), the probability of energy transfer and its efficiency will increase with the increase of acceptor content. It is consistent with the results in Figure 5g.

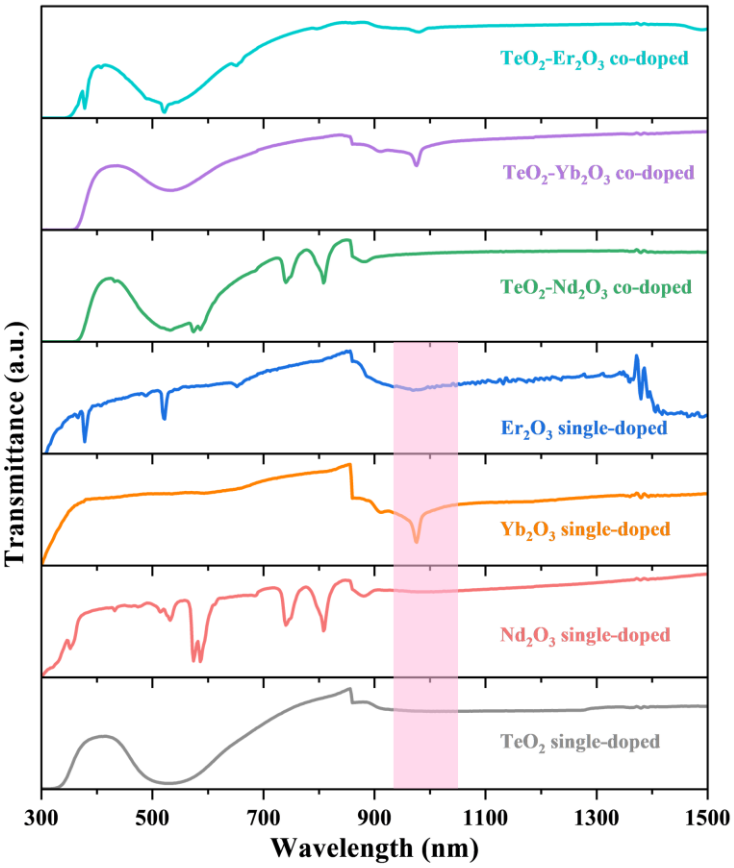

Figure S10. Transmission spectra of Te, Nd^{3+}, Yb^{3+} and Er^{3+} singly doped and Te-Nd^{3+}, Te-Yb^{3+}, Te-Er^{3+} co-doped samples at 300–1500 nm.

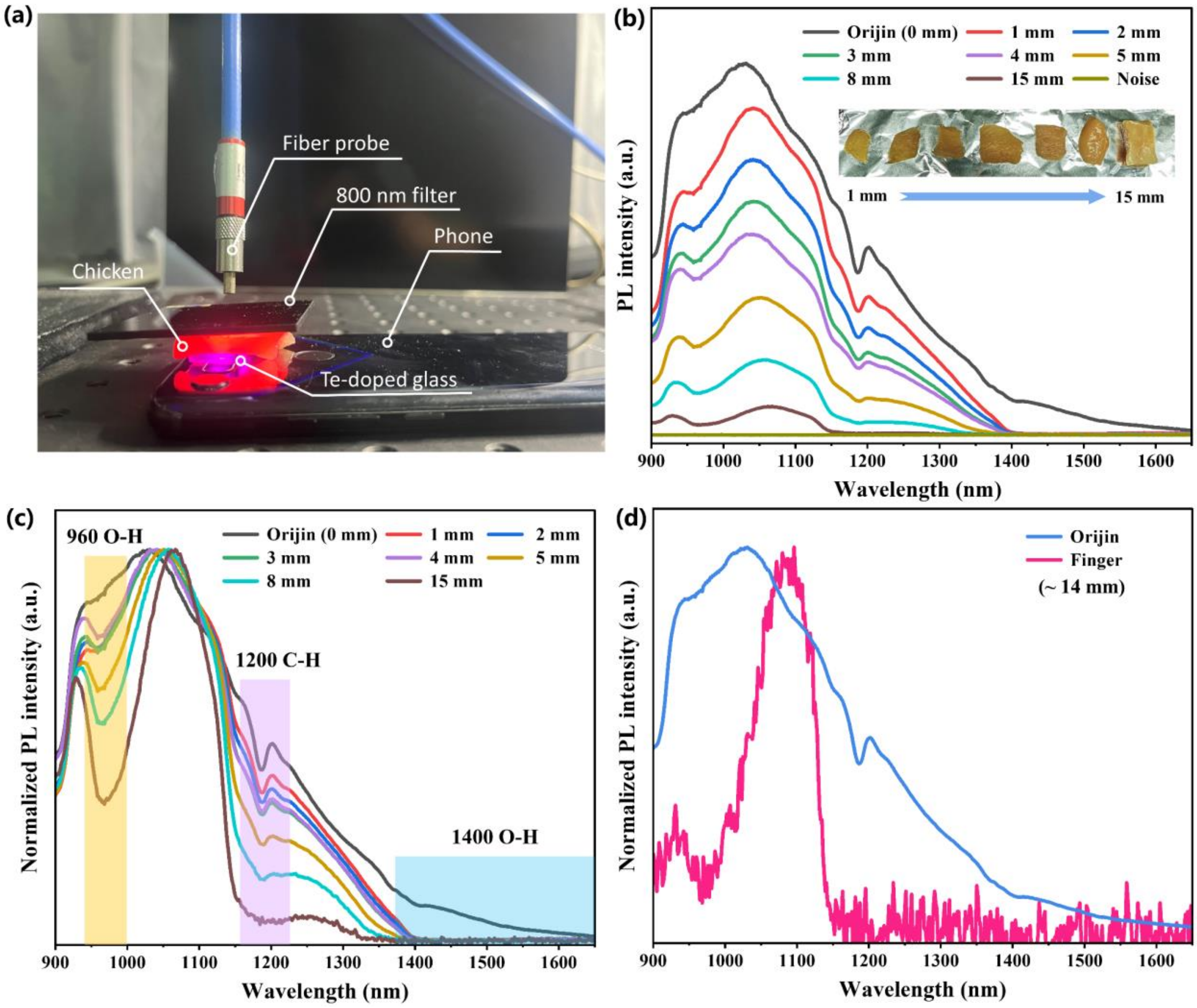

Figure S11. (a) Diagram of the actual setup used to detect the NIR light source made by Te photonic glass for penetrating the chicken breast. (b, c) The actual spectra (b) and normalized spectra (c) of the NIR light source penetrating chicken breasts with different thicknesses. The inset is the actual picture of chicken breasts. (d) The normalized spectra of NIR light before and after it penetrates the fingers.

In order to demonstrate the penetration performance of NIR light on biological tissue, chicken breast slices of different thicknesses were covered on the NIR device, and the signal was collected by using an optical fiber spectrometer. The diagram of the experimental setup and the results are shown in Figure S11a. Because O-H and C-H groups in meat tissue have strong absorption in the NIR waveband region, the collected NIR light gradually weakens as the

thickness of the chicken breast increases (Figure S11b). In particular, at the characteristic absorption positions of these two groups such as 960, 1200, and 1400 nm regions, a significant decrease can be observed (Figure S11c). In addition, when replacing the chicken breast with fingers (~14 mm thickness), a narrow NIR signals can still be collected because other spectral regions are strongly absorbed by tissues such as skin and blood (Figure S11d).

Table S1. Actual nitrogen content in glass samples doped with different AlN contents

Sample	1AlN	2AlN	3AlN	4AlN	6AlN
Nominal content (wt.%)	0.0012	0.0024	0.0037	0.0049	0.0073
Actual content (wt.%)	< 0.002	< 0.002	0.0025	0.0034	0.0053

Table S2. Potential assignment of Raman peaks for the Te-doped borate glasses

Peak position (cm^{-1})	Assignment	Reference
185	Te cluster	
214	Te_2 cluster	[1-3]
370, 428	Overtone of the fundamental mode of Te cluster	[1-3]
550	Symmetric B-O-B stretch of BO_4 units	[4, 5]
750	Symmetric B-O-B stretch of six-membered rings with two BO_4 tetrahedral	[6, 7]
905	B-O stretch of orthoborate units	[4, 6, 7]
1050	B-O-B stretch of diborate units	[4, 6, 8]

Table S3. Luminescence performance comparison of the as-prepared Te-doped borate glass with the reported rare-earth and transition-metal ions-doped NIR crystals.

Composition	λ_{ex} (nm)	λ_{em} (nm)	FWHM (nm)	Reference
$K_3LuSi_2O_7$:Eu^{2+}	460	740	160	[9]
$Y_{3-x}Ca_xAl_{5-x}Si_xO_{12}$:$Cr^{3+}$	440	760	160	[10]
K_2NaScF_6:Cr^{3+}	435	765	101	[11]
K_3ScF_6:Cr^{3+}	432	770	150	[12]
Cs_2KGaF_6:Cr^{3+}	439	782	110	[13]
$Ca_2LuHf_2Al_3O_{12}$:Cr^{3+}	460	785	145	[14]
$KAlP_2O_7$:Cr^{3+}	450	790	120	[15]
$CaLuScGa_2Ge_2O_{12}$:Cr^{3+}	465	800	150	[16]
$MgAl_2O_4$:Mn^{2+}	450	813	125	[17]
$LiInSi_2O_6$:Cr^{3+}	460	840	143	[18]
$ScTaO_4$: Cr^{3+}	516	940	186	[19]
MgO:Cr^{3+}, Ni^{2+}	455	1335	235	[20]
$LiMgPO_4$:Cr^{3+}, Ni^{2+}	450	1380	273	[21]
$Y_3Al_2Ga_3O_{12}$:Ni^{2+}	400	1450	300	[22]
Nd^{3+}-doped borate glass	808	1060	30	This work
Yb^{3+}-doped borate glass	980	990	70	This work
Er^{3+}-doped borate glass	980	1550	40	This work
Te-doped borate glass	Full visible (peak @520 nm)	1030	330	This work

Supplementary References

[1] L. Tan, Y. Fu, S. Kang, L. Wondraczek, C. Lin, Y. Yue, *Photonics Res.* **2022**, 10, 1187

[2] Q. Chen, F. Zhang, Z. Chen, J. Qiu, *J. Non-Cryst. Solids* **2017**, 458, 76.

[3] Q. Dong, K. Zhang, J. Chen, W. Chen, X. Feng, X. Li, Z. He, J. Qiu, S. Zhou, *Ceram. Int.* **2023**, 49, 22313.

[4] C. N. Santos, D. de Sousa Meneses, P. Echegut, D. R. Neuville, A. C. Hernandes, A. Ibanez, *Appl. Phys. Lett.* **2009**, 94, 151901.

[5] G. D. Chryssikos, E. Kamitsos, A. Patsis, M. Karakassides, *Mater. Sci. Eng. B* **1990**, 7, 1.

[6] D. Möncke, E. Kamitsos, D. Palles, R. Limbach, A. Winterstein-Beckmann, T. Honma, Z. Yao, T. Rouxel, L. Wondraczek, *J. Chem. Phys.* **2016**, 145.

[7] E. Kamitsos, M. Karakassides, *Phys. Chem. Glasses* **1989**, 30, 19

[8] B. Meera, A. Sood, N. Chandrabhas, J. Ramakrishna, *J. Non-Cryst. Solids* **1990**, 126, 224.

[9] J. Qiao, G. Zhou, Y. Zhou, Q. Zhang and Z. Xia, *Nat. Commun*, **2019**, 10, 5267.

[10] G. Zheng, W. Xiao, J. Wu, X. Liu, H. Masai and J. Qiu, *Adv. Sci*, **2022**, 9, e2105713.

[11] E. Song, H. Ming, Y. Zhou, F. He, J. Wu, Z. Xia and Q. Zhang, *Laser Photonics Rev.*, **2020**, 15, 2000410.

[12] H. J. Yu, J. Chen, R. Y. Mi, J. Y. Yang and Y. G. Liu, *Chem. Eng. J.*, **2021**, 417, 129271.

[13] Z. X. Wu, X. X. Han, J. Wang, Y. Y. Zhou, K. Xing, S. Cao, J. L. Zhao, B. S. Zou and R. S. Zeng, *J. Mater. Chem. C*, **2022**, 10, 10292-10301.

[14] L. Zhang, D. Wang, Z. Hao, X. Zhang, G.-h. Pan, H. Wu and J. Zhang, *Adv. Opt. Mater.*, **2019**, 7, 1900185.

[15] H. Zhang, J. Zhong, F. Du, L. Chen, X. Zhang, Z. Mu and W. Zhao, *ACS Appl. Mater. Interfaces*, **2022**, 14, 11663-11671.

[16] B. Bai, P. Dang, D. Huang, H. Lian and J. Lin, *Inorg. Chem.*, **2020**, 59, 13481-13488.

[17] E. Song, X. Jiang, Y. Zhou, Z. Lin, S. Ye, Z. Xia and Q. Zhang, *Adv. Opt. Mater.*, **2019**, 7,

1901105.

[18] X. X. Xu, Q. Y. Shao, L. Q. Yao, Y. Dong and J. Q. Jiang, *Chem. Eng. J.*, **2020**, 383, 123108.

[19] W. Shan, S. Zhang, S. Liu, S. Han, X. Li, C. Wang, C. Li, *Dalton Trans.*, **2022**, 42,16325-16335.

[20] B.-M. Liu, X.-X. Guo, L.-Y. Cao, L. Huang, R. Zou, Z. Zhou and J. Wang, *Chem. Eng. J.*, 2**023**, 452, 139313.

[21] S. H. Miao, Y. J. Liang, Y. Zhang, D. X. Chen and X. J. Wang, *Adv. Mater. Technol.*, **2022**, 7, 2200320.

[22] L. Yuan, Y. Jin, H. Wu, K. Deng, B. Qu, L. Chen, Y. Hu and R.-S. Liu, *ACS Appl. Mater. Interfaces*, **2022**, 14, 4265-4275.
